# The Treatment of Advanced Melanoma: Therapeutic Update

**DOI:** 10.3390/ijms23126388

**Published:** 2022-06-07

**Authors:** Alessia Villani, Luca Potestio, Gabriella Fabbrocini, Giancarlo Troncone, Umberto Malapelle, Massimiliano Scalvenzi

**Affiliations:** 1 Section of Dermatology, Department of Clinical Medicine and Surgery, University of Naples Federico II, Via Pansini 5, 80131 Naples, Italy; potestioluca@gmail.com (L.P.); gafabbro@unina.it (G.F.); scalvenz@unina.it (M.S.); 2 Department of Public Health, University Federico II of Naples, 80131 Naples, Italy; giancarlo.troncone@unina.it (G.T.); umberto.malapelle@unina.it (U.M.)

**Keywords:** melanoma, metastatic melanoma, targeted therapy, immune checkpoint inhibitors, vaccines, small molecules

## Abstract

Cutaneous melanoma is the main cause of death for skin cancer. The majority of patients with a diagnosis of melanoma have localized disease, which can be successfully treated with surgical treatment. However, the surgical approach is not curative for advanced melanoma (AM). Indeed, the management of AM is still challenging, since melanoma is the solid tumor with the highest number of mutations and cancer cells have the capacity to evade the immune system. In the past, the treatment of AM relied on chemotherapeutic agents, without showing efficacy data. Recent knowledge on melanoma pathogenesis as well as the introduction of immunotherapies, targeted therapies vaccines, small molecules, and combination therapies has revolutionized AM management, showing promising results in terms of effectiveness and safety. The aim of this review is to assess and to discuss the role of emerging therapies for AM management in order to obtain a complete overview of the currently available treatment options and future perspectives.

## 1. Introduction

The incidence of primary cutaneous melanoma (CM) is increasing every year, with up to 132,000 diagnosis annually [1]. Moreover, CM is the main cause of death for skin cancer [1,2] Tumor stage at the diagnosis is the main predictor of survival rate, accounting for 98.3% at 5 years for localized melanoma and 16% for metastatic disease [3]. Tumor thickness (Breslow score), lymph node involvement, and the presence of metastasis are at the basis of melanoma staging, dividing melanoma severity into four stages: stage I and II referring to localized disease, stage III and stage IV characterized by metastasis to the local lymph nodes or distant metastasis, respectively [1,3,4].

The majority of patients with a diagnosis of melanoma have localized disease, successfully treated with surgical treatment [5]. Sentinel lymph node biopsy is mandatory if CM is greater than 0.8 mm thick or thinner than this but ulcerated [5]. The removal of the remaining lymph nodes in the area is sometimes performed if the sentinel lymph node is positive [5]. However, surgical treatment is not curative for AM [3,5,6]. Treatment of AM is still challenging since melanoma is the solid tumor with the highest number of mutations and cancer cells have the capacity to evade the immune system [7]. Historically, AM management relied on chemotherapeutic agents, however without ever showing efficacy data [3,5,6]. Recent knowledge on melanoma pathogenesis as well as the introduction of immunotherapies, targeted therapies vaccines, small molecules, and combination therapies has revolutionized AM management [4].

The objective of this review is to evaluate and discuss the role of emerging therapies for AM management in order to obtain a complete overview of the currently available treatment options and future perspectives.

## 2. Material and Methods

A literature search was carried out on the Pubmed, Embase, and Cochrane Skin, clinicaltrials.gov databases (until 10 March 2022) using the following research terms: “cutaneous melanoma”, “checkpoint immunotherapy”, “targeted therapies”, “BRAF inhibitors”, “MEK inhibitors”, “immunotherapy”, “vemurafenib”, “dabrafenib”, “ipilimumab”, “nivolumab”, “pembrolizumab”, “trametinib”, “binimetinib”, “sequential treatment”, “cytokines”, “vaccines”, “anti-vascular endothelial growth factor”, “inhibitory molecules”, and “T Cell Agonists”.

Analyzed articles included reviews, metanalyses, clinical trials (CT), real-life studies (RLS), case series, and reports. The most relevant manuscripts were considered. A revision of the bibliography was also performed to include articles that could have been missed. Assessment of treatment efficacy was made through overall survival (OS), progression-free survival (PFS), recurrence-free survival (RFS), disease-free survival (DFS), durable response rate (DRR), and overall response rate (ORR). Articles regarding treatments for non-advanced CM were excluded. Thus, the research was refined by reviewing the abstracts and texts of selected articles. Only English language manuscripts were considered, while French, German, and Spanish language works were excluded. This article is based on previously conducted studies and does not contain any studies with human participants or animals performed by any of the authors.

## 3. Results

Data regarding the efficacy and safety of drugs currently approved for the management of metastatic or unresectable melanoma and as adjuvant treatment of patients with melanoma and lymph node involvement are reported in Table 1 and Table 2, respectively.

### 3.1. Immune Checkpoint Inhibitors

Immune checkpoint inhibitors are drugs which act through the blockage of small proteins produced by immune cells and cancer cells called “*checkpoints*” [8,9]. In particular, programmed cell death protein-1 (PD-1) and cytotoxic T lymphocyte–associated antigen-4 (CTLA-4) are *checkpoints* which downregulate T cell activation, produced by cancer cells in order to escape from immunity system, producing immune tolerance [8,9]. The binding of PD-1, also known the cluster of differentiation 279 (CD279), which is expressed on the surface of monocytes, T cells, and B and NK cells, to its ligand PDL-1 promotes the apoptosis of T cells and activates the regulatory T cells, thus preventing the inflammation pathway [8,9]. CTLA-4, also known as CD152, is constitutively expressed in regulatory T cells and inhibits the activation of T cells [8,9]. Targeting these pathways is a valuable weapon to reduce melanoma immune escape.

#### 3.1.1. Pembrolizumab

Pembrolizumab is an anti-PD-1 molecule currently approved for the management of patients with metastatic or unresectable melanoma and for the adjuvant treatment of patients with melanoma and with lymph node involvement at a dosage of 200 mg every three week (Q3W) or 400 mg Q6W [10]. Several clinical trials have shown its efficacy and safety in the treatment of metastatic or unresectable melanoma and as adjuvant therapy [10,11,12,13,14,15,16,17,18,19,20,21,22]. The effectiveness of pembrolizumab was firstly reported in a randomized (1:1:1), open-label, multicenter, controlled trial (KEYNOTE-006) [10,15,16,17]. A total of 834 patients were randomized to receive pembrolizumab 10 mg/kg intravenously Q2W, 10 mg/kg intravenously Q3W, or ipilimumab 3 mg/kg intravenously Q3W. Inclusion criteria were: unresectable or metastatic melanoma; no prior ipilimumab; and <1 prior systemic treatment for metastatic melanoma. The efficacy was assessed through PFS, OS, and ORR, evaluated at week 12 and, thereafter, every 6 weeks up to week 48, followed by every 12 weeks thereafter. A statistically significative improvement in OS and PFS was observed in patients who received pembrolizumab compared to ipilimumab. ORR was assessed in 91 (response durations ranged from 1.4 to 8.1 months) and 94 (response durations ranged from 1.4 to 8.2 months) patients who received pembrolizumab 10 mg/kg Q3W and 10 mg/kg Q2W, respectively. Regarding drug safety, 9% of patients permanently discontinued pembrolizumab due to adverse reactions, with colitis representing the main one (1.4%), while treatment interruption was observed in 21%, mainly for diarrhea (2.5%). Safety profile was similar in both pembrolizumab treatment groups. Fatigue (28%) and hyperglycemia (45%) were the main AEs laboratory abnormalities reported.

KEYNOTE-002 [10,11] was a multicenter, randomized, controlled trial, which enrolled 540 patients divided in three groups (1:1:1). Group 1 received pembrolizumab 2 mg/kg Q3W; Group B, 10 mg/kg Q3W; and Group C, chemotherapy ((dacarbazine (26%), temozolomide (25%), and carboplatin AUC intravenously plus paclitaxel 225 mg/m^2^ (25%), paclitaxel (16%), or carboplatin AUC (8%)). Inclusion criteria were represented by (a) patients who failed two or more doses of ipilimumab and a BRAF or MEK inhibitor (if BRAF V600 mutation-positive); or (b) disease progression within 24 weeks following the last dose of ipilimumab. Patients with uveal melanoma and active brain metastasis were excluded. Tumor status was evaluated at week 12 and every 6 weeks up to week 48, followed by every 12 weeks thereafter. The efficacy was evaluated through PFS, OS, and ORR. A statistically significative improvement in PFS was observed in patients treated with pembrolizumab compared to control group. No statistically significant differences in OS were observed among the groups. An ORR was assessed in 38 (response duration ranged from 1.3+ to 11.5+ months) and 46 (response durations ranged from 1.1+ to 11.1+ months) patients in the 2 mg/kg and 10 mg/kg group, respectively. Regarding safety, no statistically significant differences were observed in patients who received pembrolizumab 2 mg/kg compared to the 10 mg/kg group. Pruritus was the most common AE reported (28%) as well as hyperglycemia (49%) for laboratory anomalies. Permanent treatment interruption was observed in 12% of patients (mainly for diarrhea, dyspnea, and maculo-papular rash), while 14% of subjects temporarily interrupted pembrolizumab due to AEs.

The effectiveness and safety of pembrolizumab was also shown in a phase Ib (KEYNOTE-001) trial, which enrolled 655 patients with melanoma [23]. Among these, 151 were treatment-naive and 496 had been previously treated. Globally, an ORR of 41% can be observed, 52% in the treatment-naive cohort.

A multicenter, randomized (1:1), double-blind, placebo-controlled trial (KEYNOTE-054) investigated the efficacy of pembrolizumab as adjuvant treatment of resected melanoma (stage IIIA-IIIB-IIIC) [10,19,20,21]. Recurrence-free survival (RFS), defined as the time between the randomization date and the date of first recurrence or death, was evaluated as the main effectiveness outcome. Pembrolizumab 200 mg Q3W was administered to 514 subjects while the remaining ones received placebo. A statistically significant improvement in RFS was reported in patients undergoing treatment with pembrolizumab compared to placebo. The study population characteristics were: a median age of 54 years (range: 19 to 88), 25% age 65 or older; 62% male; and 94% ECOG PS of 0 and 6% ECOG PS of 1. Sixteen percent had stage IIIA, 46% had stage IIIB, 18% had stage IIIC (1–3 positive lymph nodes), and 20% had stage IIIC (≥4 positive lymph nodes). Serious AEs were reported in 25% of patients treated with pembrolizumab, with 14% of subjects permanently interrupting treatment, principally for pneumonitis (1.4%), and 19% temporarily suspending treatment, mainly due to diarrhea, 2.4%. Two patients undergoing treatment with pembrolizumab died (one presenting eosinophilia and systemic symptoms and one autoimmune myositis with respiratory failure). Other studies investigating the effectiveness of pembrolizumab as adjuvant treatment in resected high-risk stage II melanoma are still ongoing [22]. The effectiveness and safety of pembrolizumab has been reported also in real-life studies [12].

Finally, a recent study investigating the efficacy and safety of pembrolizumab in patients older than 85 years showed that high risk of toxicity and impaired autonomy may be associated with pembrolizumab use in elderly patients [13]. In this *scenario*, a phase I study showed that pembrolizumab at a labelled dosage plus reduced-dose ipilimumab may be a valuable treatment option for metastatic melanoma [14].

#### 3.1.2. Nivolumab

Nivolumab is an immunotherapy medication approved for the treatment of metastatic or unresectable melanoma and as adjuvant therapy in patients with lymph node involvement, which blocks programmed cell death 1 ligand 1 (PD-L1) from binding to PD-1, allowing the immune response to cancer cell. It is approved as single agent administered as an intravenous infusion at dosage of 240 mg Q2W or 480 mg Q4W or in combination with ipilimumab (nivolumab 1 mg/kg followed by ipilimumab 3 mg/kg Q3W up to four doses followed by nivolumab as single agent) [24].

The efficacy and safety of nivolumab in previously treated (ipilimumab and BRAF inhibitor, if BRAF V600 mutation was positive) metastatic melanoma was evaluated in a multicenter, randomized (2:1) trial (CHECKMATE-037), which enrolled 450 subjects [24,25,26]. Patients received 3 mg/kg of nivolumab Q2W or chemotherapy (dacarbazine, carboplatin + paclitaxel). Patients were evaluated at week 9 and every 6 weeks for the first year, then every 12 weeks. A total of 120 patients received nivolumab for at least 6 months. Among these, an ORR of 32% was reported with 4 complete responses and 34 partial responses. The OS in nivolumab-treated patients was 15.7 months, while in patients treated with chemotherapy it was 14.4 months. As regards the safety, 9% of patients discontinued treatment due to multiple AEs and 26% delayed the administration for AEs. Rash was the AE most frequently reported (21%).

CHECKMATE-066 evaluated the efficacy of nivolumab 3 mg/kg Q2W compared to dacarbazine 1000 mg/m^2^ Q3W in a multicenter, double-blind randomized (1:1) trial, including patients with untreated metastatic melanoma. The effectiveness was evaluated at week 9 and every 6 weeks up to 1 year and every 12 weeks thereafter. Main outcomes were the OS, PFS, and ORR. A total of 418 subjects were enrolled; among these 210 received nivolumab and 208 dacarbazine. A statistically significant improvement in OS was observed in nivolumab group. The safety of nivolumab was suggested in CHECKMATE-066 as well. Indeed, only 7% and 26% of patients permanently or temporarily discontinued drug administration due to AE, respectively. Gamma-glutamyl transferase increase (3.9%) and diarrhea (3.4%) were the most common Grade 3 and 4 AEs reported.

Similarly, a multicenter, randomized (1:1:1) double-blind trial (CHECKMATE-067) compared the effectiveness of nivolumab (316 patients, nivolumab 3 mg/kg Q2W) in untreated patients with unresectable or metastatic melanoma to patients treated with nivolumab + ipilimumab (314 patients, nivolumab 1 mg/kg with ipilimumab 3 mg/kg Q3W, followed by nivolumab 3 mg/kg as a single agent Q2W after four doses) and only ipilimumab (314 patients, ipilimumab 3 mg/kg Q3W for four doses, followed by placebo Q2W) [24,27,28]. Inclusion criteria were: patients who completed adjuvant or neoadjuvant treatment at least 6 weeks before the randomization, but not treated with anti-CTLA-4. A total of 945 subjects were randomized. A statistically significant improvement was shown in OS and PFS for patients receiving nivolumab as single therapy or combined with ipilimumab compared to the ipilimumab group. Regarding the safety, in CHECKMATE-067, AEs led to nivolumab discontinuation in 18% of cases in patients treated with nivolumab as single agent, whereas 36% of patients delayed the administration in the same cohort. Diarrhea was the most common serious AE reported both in ipilimumab plus nivolumab and nivolumab as single agent group (13% and 2.2%).

As regards adjuvant treatment, CHECKMATE-238, a randomized, double-blind, randomized (1:1) study, which enrolled 906 patients receiving 3 mg/kg of nivolumab Q2W or ipilimumab 10 mg/kg Q3W in four doses every 12 weeks [24,29,30]. RFS improvement for patients receiving nivolumab was statistically significantly higher compared to the ipilimumab group. About 9% of patients in the nivolumab group suspended treatment while 28% delayed the administration.

#### 3.1.3. Ipilimumab

Ipilimumab is an anti-CTLA-4 antibody that blocks CTLA-4 activity, thus promoting T cells function and growth. It is approved at a dosage of 3 mg/kg Q3W for maximum four doses [31]. Its effectiveness and safety have been reported in a randomized (3:1:1), double-blind study (MDX010-20), which enrolled 676 patients with unresectable or metastatic melanoma previously treated with chemotherapeutic agents [31,32]. Among these, 403 received ipilimumab plus an investigational peptide vaccine with an incomplete Freund’s adjuvant (gp100), 137 ipilimumab as single agent, and 136 gp100 as a single agent. Clinical assessment was evaluated at week 12 and 24, and every 3 months thereafter. The ORR was 5.7%, 10.9%, and 1.5% in the ipilimumab+gp100, ipilimumab, and gp100 arm, respectively. Regarding safety, fatigue (41%) was the most common AE reported in the group treated with ipilimumab as a single agent, followed by diarrhea (32%) and pruritus (31%).

CA184-029, a randomized (1:1), double-blind, placebo-controlled trial, evaluated the effectiveness of ipilimumab 10 mg/kg Q3W up to 4 doses compared to placebo, followed by ipilimumab 10 mg/kg or placebo every 12 weeks up to week 156 as adjuvant treatment. Disease assessment was performed every 12 weeks up to week 156 then every 24 weeks thereafter. A total of 951 patients were enrolled. Among these, 475 received ipilimumab. RFS was significantly higher for patients receiving ipilimumab compared to placebo. Treatment was discontinued in 52% of patients due to AEs. A rash was the most common AE reported (50%).

### 3.2. Targeted Therapies

BRAF is a serine/threonine protein kinase which acts through the activation of the mitogen-activated protein (MAP) kinase/ERK-signaling pathway, leading to the evasion of senescence, apoptosis and immune response, unchecked replicative potential, angiogenesis, tissue invasion, and metastasis. BRAF mutations are present in about 50% of melanomas. In particular, over 90% of the mutations are at codon 600, with over 90% represented by a single nucleotide mutation resulting in substitution of glutamic acid for valine (BRAFV600E: nucleotide 1799 T  >  A; codon GTG  >  GAG) [33]. Drugs selectively inhibiting BRAF have shown excellent results in the treatment of AM.

Combining BRAF and MEK inhibition is a new strategy for the management of melanoma. Indeed, combination therapy showed significantly better results compared to monotherapy [33].

#### 3.2.1. Vemurafenib

Vemurafenib is a targeted therapy approved for the management of patients with metastatic or unresectable melanoma with BRAF V600E mutation at the dosage of four tablets of 240 mg every 12 h [34,35].

Its efficacy has been evaluated in a randomized-controlled trial (TRIAL 1) involving 675 patients with unresectable or metastatic melanoma BRAF V600E mutation-positive receiving vemurafenib 960 mg twice a day (337) or dacarbazine (338). A statistically significant improvement in OSS and PFS was reported in the vemurafenib group compared to dacarbazine group with an ORR of 48.4% AND 5.5% in vemurafenib and dacarbazine group, respectively. As regards the safety, arthralgia was the main AE reported (53%) in vemurafenib-treated group, followed by alopecia (45%) and fatigue (38%). Patients who permanently discontinued treatment for AE represented 7% [34,35].

TRIAL 2 evaluated the use of vemurafenib 960 mg twice a day in 132 patients previously treated with systemic therapy for metastatic melanoma BRAF V600E mutated. The ORR assessed was 52% with a mean duration of 6.5 months. In Trial 2, only 3% of patients discontinued the study due to AE [34,35].

Finally, TRIAL 3 evaluated the effectiveness of vemurafenib 960 mg twice daily in patients with metastatic melanoma BRAF V600E mutation-positive melanoma, with brain metastasis 3. A total of 146 patients were enrolled and divided in two groups (group A, 90 patients: no prior local therapy for brain metastases; group B, 56 patients: at least one prior local therapy for brain metastases). An ORR of 18% was reported in both groups with a median duration of the response of 4.6 and 6.6 months in group A and B, respectively) [34,35].

#### 3.2.2. Dabrafenib

Dabrafenib is a kinase inhibitor approved for the treatment of metastatic or unresectable melanoma with a BRAF V600E or V600K mutation and as an adjuvant treatment of subjects with melanoma plus lymph node involvement (BRAF V600E- or V600K-mutated) [36].

A multicenter, randomized (3:1) study, which enrolled 250 patients with previously untreated metastatic or BRAF V600E-mutated unresectable melanoma, evaluated the efficacy of dabrafenib 150 mg twice a day (187) compared to dacarbazine (63) in BREAK-3 study. A statistically significant improvement in PFS was reported in the dabrafenib group. Hyperkeratosis (37%), followed by headache (32%), was the main AE reported. Moreover, 3% of patients discontinued the treatment due to AEs [37,38].

The use of dabrafenib 150 mg twice a day was also evaluated in metastatic melanoma BRAF V600E mutated with brain metastasis. In particular, 74 patients (group A) were not previously treated with local therapy for brain metastasis, while 65 (Group B) received at least one local therapy, an overall intracranial response rate of 18% with a mean duration of 4.6 months was reported in both groups [36].

The association of dabrafenib (150 mg twice daily) and trametinib (2 mg once daily) in unresectable or metastatic BRAF V600E- or V600K-mutated cutaneous melanoma was evaluated in two randomized controlled trials (COMBI-d study and COMBI-v study) [36]. A total of 423 patients were randomized in the COMBI-d study, with 211 and 212 receiving dabrafenib plus trametinib and dabrafenib plus placebo, respectively. In the COMBI-v study, 352 patients received dabrafenib plus trametinib and 352 vemurafenib. A statistically significant improvement in OS and PFS was reported in both studies. Pyrexia, rash, arthralgia, chills, and headaches were the main AEs reported in both studies. Treatment discontinuation was reported in 11% of patients receiving dabrafenib plus trametinib [36].

Finally, COMBI-AD evaluated the use of dabrafenib (150 mg twice a day) plus trametinib (2 mg once a day) in 438 patients compared to placebo (432 patients) as adjuvant treatment in patients with the pathologic involvement of regional lymph node. A statistically significant improvement of relapse-free survival was reported in patients receiving dabrafenib + trametinib compared to placebo. As regards the safety, pyrexia was the most commonly occurring AE (55%), followed by fatigue (51%) and nausea (45%). Moreover, 25%, 35%, and 66% of patients discontinued, reduced, or interrupted the dose, respectively [39].

#### 3.2.3. Encorafenib

Encorafenib is a kinase inhibitor, approved for the management of unresectable or metastatic melanoma with BRAF V600E or BRAF V600K mutation at the dosage of 450 mg (six 75 mg capsules) once a day in combination with binimetinib 45 mg twice a day [40]. A total of 577 patients were divided to receive encorafenib plus binimetinib (192), encorafenib as single agent (194), and vemurafenib (191). Encorafenib plus in binimetinib showed a statistically significant improvement in PFS compared to vemurafenib. The most common AE reported in patients treated with encorafenib plus binimetinib was fatigue (43%). Encorafenib administration was interrupted in 30% of patients receiving encorafenib plus binimetinib, while 14% of patients needed dose reduction and 5% permanently suspended the treatment [40].

#### 3.2.4. Trametinib

Trametinib is a kinase inhibitor targeting MEK1 and MEK2, approved for the management of metastatic or BRAF V600E- or BRAF V600K-mutated unresectable melanoma or as adjuvant treatment at an oral dosage of 2 mg a day [41]. METRIC study evaluated the efficacy of trametinib as single agent in a randomized (2:1), multicenter trial, which enrolled 332 patients with metastatic or V600E- or V600K-mutated unresectable melanoma. Among these, 214 received trametinib 2 mg once a day and 108 dacarbazine or paclitaxel. A statistically significant improvement in PFS was observed in patients treated with trametinib. As regards the safety of trametinib, 9% of patients discontinued the treatment, most commonly for decreased left ventricular ejection fraction, renal failure, rash pneumonitis, and diarrhea. Moreover, 27% of patients reduced the dosage due to AEs [42]. The effectiveness and safety of trimetinib plus dabrafenib was reported in COMBI-d and COMBI-AD study as well.

#### 3.2.5. Binimetinib

Binimetinib is a MEK inhibitor approved for the management of metastatic or BRAF V600E-mutated unresectable melanoma at the dosage of 45 mg twice a day in combination with encorafenib [43].

A randomized (1:1:1) phase III study evaluated the efficacy of binimetinib plus encorafenib in patients with metastatic or unresectable melanoma with BRAF V600E or V600K mutation (Study CMEK162B2301). Among the enrolled patients, 192 received binimetinib plus encorafenib, 194 encorafenib, and 191 vemurafenib. A statistically significant improvement in PFS and OS in patients treated with binimetinib plus encorafenib was shown [43]. Moreover, in Study CMEK162B2301, Part 2 evaluated the contribution of bimetinib to the bimetinib plus encorafenib association [43]. Fatigue, nausea, and diarrhea were the main AEs reported. A total of 258 patients receiving combination treatment were compared to 280 patients receiving only encorafenib, showing a significative improvement in PFS for the combo treatment group [43].

### 3.3. Other Emerging Therapies

#### 3.3.1. Sequential Treatment

Given the high effectiveness of both targeted therapies and immunotherapies, the combination of these drugs as an effective weapon for patients with melanoma BRAF V600-mutated was a logical consequence. However, acquired resistance to both targeted therapy and immunotherapy led to the need for several studies investigating different combination therapies. Among these, the sequence of targeted and immune checkpoint therapy showed promising results in terms of safety and efficacy in patients with metastatic or unresectable melanoma with a BRAF mutation. The use of dabrafenib and trametinib followed by ipilimumab plus nivolumab or the use of nivolumab plus ipilimumab followed by dabrafenib plus trametinib (DREAMseq) is currently being investigated in a phase III trial. The study is ongoing, and results are not available [44]. Similarly, ImmunoCobiVem, a phase II trial evaluating the use of atezolizumab (Arm a) or vemurafenib and cobimetinib (Arm B) after a 3-month run-in period with vemurafenib plus cobimetinib is ongoing. A total of 176 participants with metastatic or BRAF-mutated unresectable melanoma were enrolled. Results are not available [45]. Finally, the sequential use of encorafenib plus binimetinib, followed by ipilimumab plus nivolumab; the same therapies in reverse order; and encorafenib and binimetinib, followed by ipilimumab plus nivolumab is currently being investigated in a randomized phase II trial involving 251 participants. The study is still ongoing [46].

#### 3.3.2. Vaccines

Talimogene laherparepvec (T-VEC) is an oncolytic immunotherapy derived from herpes simplex virus type 1. It acts by selectively replicating within tumors, leading to a systemic antitumor immune response through the production of granulocyte macrophage colony-stimulating factor (GM-CSF) [47] T-VEC is the only oncolytic viral therapy tested in a randomized clinical study.

The effectiveness of T-VEC was firstly showed in a phase II trial involving 50 patients with unresectable regional disease (*n* = 10), skin- or lymph node-only metastases (*n* = 16) and visceral metastases (*n* = 24) receiving T-VEC Q3W. An ORR of 26% was reported. Moreover, both injected and uninjected lesions showed a clinical response as well as disease progression prior to response (“pseudo-progression”) was assessed in many cases [48].

A phase III clinical trial enrolling 436 patients affected by unresectable injectable melanoma randomized to receive intralesional T-VEC (*n* = 295) or subcutaneous GM-CSF (*n* = 141) showed T-VEC as an effective weapon in melanoma management. Indeed, a statistically significant improvement of disease was reported in T-VEC cohort compared to GM-CSF group (ORR: 26.4%, *p* < 0.001). Fatigue, chills and pyrexia were the main AEs reported [47]. Similarly, OPTiM, a phase III randomized (2:1) study showed an ORR significantly higher in patients receiving T-VEC compared to GM-CSF (26.4 vs. 5.7%, *p* < 0.0001). The only AE reported in more than two patients was cellulitis [49].

#### 3.3.3. Cytokines

The use of engineered cytokine may be a valuable weapon for the management of melanoma.

***L19IL2 (Darleukin)***

Darleukin (L19IL2) is a fully human immunostimulatory product consisting of the fusion of the human L19 antibody and IL2. Its efficacy and safety has been evaluated in a phase II trial enrolling 69 patients with metastatic melanoma randomized to receive dacarbazine or dacarbazine plus darleukin at two different dosages (24, 23 and 22 patients, respectively), A significant result in terms of ORR was assessed in patients receiving L19IL2 plus dacarbazine compared to dacarbazine as single agent [50]. Currently, a study (Neo-DREAM) investigating the effectiveness of neoadjuvant Intratumoral Darleukin/Fibromun (L19IL2 + L19TNF) in patients with AM (Stage IIIB/C) is ongoing [33].

***Bempegaldesleukin (BEMPEG; NKTR-214)***

Bempegaldesleukin is an engineered IL-2R agonist which reduces IL-2 binding to CD25 over CD122/CD132, stimulating an antitumor immune response [51].

The effectiveness and safety of bempegaldesleukin plus nivolumab was evaluated in a phase II trial (PIVOT-02) involving 38 patients with previously untreated metastatic melanoma. At a 29 months follow-up, an ORR of 52.6% was observed, with a CR of 34.2% [51].

A phase III, randomized, open-label trial (PIVOT IO 001) evaluating the efficacy and safety of bempegaldesleukin plus nivolumab or nivolumab in monotherapy in patients with previously untreated, unresectable, or metastatic melanoma is ongoing [52]. Results are not yet available [52].

#### 3.3.4. Intravenous Oncolytic Virus

Among the emerging treatment of cancer, an oncolytic adenovirus-based therapy showed promising results. The virus can selectively infect tumor cells, leading to oncolysis and release of new viruses which induce an immune response against the tumor. ICOVIR-5 is derived from the oncolytic adenovirus Ad-Δ24 arginine-glycine-aspartic acid [52]. A phase I trial, including 12 patients with uveal and cutaneous metastatic malignant melanoma, showed that the administration of adenovirus ICOVIR-5 is well tolerated [52]. Future studies will allow the evaluation of the effectiveness of this treatment option.

#### 3.3.5. Anti-Vascular Endothelial Growth Factor

Angiogenesis is a relevant target for melanoma management. Several angiogenesis inhibitors are currently being tested in both metastatic and adjuvant melanoma [52]. Among these, bevacizumab, a systemic anti-vascular endothelial growth factor, showed promising results [52].

A phase I trial reported the safety of Bevacizumab and imatinib in patients with metastatic melanoma [52]. A multicenter phase II trial assessed the effectiveness and safety of carboplatin-paclitaxel plus bevacizumab as first-line therapy in patients’ unresectable metastatic melanoma. A total of 50 patients were enrolled. An ORR of 34% was showed that peripheral neuropathy, fatigue, alopecia, and gastrointestinal disorders were the main AEs reported [53]. Finally, AVAST-M, a multicenter, randomized, controlled phase III trial evaluating the effectiveness of bevacizumab as adjuvant treatment in patients with melanoma is ongoing [54].

#### 3.3.6. Targeting Inhibitory Molecules: CSF1Ri and IDOi

Colony-Stimulating Factor 1 Receptor Inhibitors

Colony-stimulating factor 1 (CSF1) is a monocyte/macrophage differentiation regulator factor, expressed in human melanoma, which sustains the protumorigenic mechanism of tumor-associated macrophages. Targeting the CSF1 receptor (CSF1R) may be a new strategy in melanoma management.

Several CSF1R Inhibitors (pexidartinib, PLX7486, ARRY-382, JNJ-40346527, BLZ945, emactuzumab, AMG820, IMC-CS4) are currently under investigation [55].

A phase I trial, which enrolled 26 patients with anti-PD-1/PD-L1-resistant melanoma (*n* = 12), non-small cell lung cancer (*n* = 1) and renal cell carcinoma (*n* = 13), evaluated the safety of the CD40 agonist APX005M (sotigalimab) and CSF1R inhibitor (cabiralizumab) with or without nivolumab. An increase of lactate dehydrogenase (*n* = 26), creatine kinase (*n* = 25), and aspartate aminotransferase (*n* = 25) were the most common AEs observed [55].

Indoleamine 2,3-Dioxygenase 1 Inhibitors

Indoleamine 2,3-dioxygenase 1 (IDO1) is an endogenous mechanism of acquired peripheral immune tolerance. Several cancers showed an increased IDO expression, associated with negative prognostic factors [55]. In melanoma, IDO1 expression was found to be increased in different stages of melanoma development, progression, and BRAF inhibitors resistance [55]. A phase I study investigated the safety of navoximod (an IDO1 inhibitor) plus atezolizumab in 158 patients with advanced cancer. Among these, four (3%) were affected by melanoma. Fatigue (22%), rash (22%), and chromaturia (20%) were the most common AEs reported. An acceptable safety, tolerability, and pharmacokinetics profile was reported [56]. A two-step clinical phase I/II trial involving 30 patients with metastatic melanoma evaluated the effectiveness and safety of a PD-L1/IDO peptide vaccine plus Nivolumab. An ORR of 80% was reported. The safety profile was similar to nivolumab in monotherapy [57].

Finally, the effectiveness of epacadostat (IDO inhibitor) and pembrolizumab was shown in a phase III, a randomized study on 706 patients with unresectable melanoma (ECHO-301/KEYNOTE-252). Among these, epacadostat plus pembrolizumab or placebo plus pembrolizumab was administered to 354 and 352 patients, respectively. However, no significant differences were found between the two groups for PFS and OS [58].

#### 3.3.7. T Cell Agonists

T cell agonists may be a potential target in melanoma management.

Toll-Like Receptors

The recognition of pathogen-associated molecular patterns and damage-associated molecular patterns by Toll-like receptors (TLRs) leads to the activation of the immune response, with pro-inflammatory cytokine release, phagocytosis, and antigen presentation. TLRs are normally expressed in keratinocytes and melanocytes. The potential effectiveness of tilstolimod, a TLR9 agonist, was suggested in a phase I trial, which enrolled three patients with PD-1 refractory metastatic melanoma [59].

OX40

Agonistic anti-OX40 antibodies showed an anti-tumor activity, associated with the infiltration and the proliferation of T cells and effector T cells at tumor sites, respectively [60]. A phase I trial evaluated the tolerability of ivuxolimab, a fully human immunoglobulin G2 agonistic monoclonal antibody specific for OX40, in a cohort of 52 patients with advanced cancer (melanoma: 2) [61]. A preliminary antitumor activity and the tolerability of ivuxolimab was reported. Similar results were reported in a phase I trial assessing the safety of MEDI0562, an agonistic humanized monoclonal antibody which specifically binds to the costimulatory molecule OX40 [62].

Glucocorticoid-Induced Tumor Necrosis Factor Receptor Family-Related Protein

Glucocorticoid-induced tumor necrosis factor receptor-related protein (GITR) is a promising target for immunotherapy, due to its capacity to promote immune response enhancing effector T cell functions and reducing regulatory T cell suppression [59].

A phase I trial reported the safety profile of TRX518, a fully humanized monoclonal antibody that triggers human GITR pathway, as monotherapy in 43 patients with advanced cancer (melanoma: 6). However, a substantial clinical response was not seen [59].

A phase I trial investigating TRX518M, a GITR pathway stimulator, in monotherapy or TRX518 plus gemcitabine, pembrolizumab, or nivolumab is ongoing [59].

4-1BB (CD137, TNFSFR9)

4-1BB is a costimulatory receptor expressed on immune cells, which causes the proliferation of effector T cells and cytokine release. A phase I study reported the tolerability of urelumab and utomilumab, two 4-IBB agonists [59].

CD27 (TNFRSF7)

CD27, also known TNFRSF7, plays a key role in T-cell activation, enhancing T-cell proliferation and differentiation. Varlilumab, a CD27 agonistic antibody, showed promising efficacy in several cancers. Current studies evaluating the combination of varlilumab plus nivolumab or atezolizumab are ongoing [59].

Adoptive T Cell Therapy

Adoptive T Cell Therapy may be a valuable option in patients with AM resistant to approved therapies [63]. It is a strategy of immunotherapy where T cells are genetically modified in order to act against cancer cells. The combination of adoptive T cell therapy with conditioning chemotherapy and high-dose IL2 showed a 55% ORR in a cohort of 101 patients with previously treated metastatic melanoma [63]. However, main limitations of this strategy include the technologies required, costs, and time required. Trials investigating the use of adoptive T cell therapy plus checkpoint inhibitors are ongoing [64].

## 4. Discussion

Recent knowledge on cutaneous tumors pathogenesis led to the development of effective and selective therapies [65]. In particular, new therapeutic approaches have revolutionized the treatment of unresectable or metastatic melanoma [64]. Indeed, the management of AM is challenging since melanoma is the solid tumor with the highest number of mutations and cancer cells have the capacity to evade the immune system [7]. In the past decade, the treatment and survival for patients with AM has improved dramatically, since several therapies, such as BRAF, CTLA4 and PD1 inhibitors, have been approved for the management of metastatic or unresectable melanoma, showing promising results in terms of effectiveness and safety [64,66]. Moreover, the association of targeted therapies and immunotherapies was shown to be a valid weapon in melanoma management. Other therapeutic options (e.g., T cell agonists, intravenous oncolytic virus, vaccines, cytokines, etc.) are currently under investigation. However, real-life data are needed to assess the effectiveness and safety of these therapies in a real-life setting [64,66].

The scenario of adjuvant or neoadjuvant treatment is changing as well, with the development of treatments with a higher probability of inducing complete and durable remission. It is probable that the association of surgical and medical interventions will be the main strategy in improving long-term outcomes for patients with AM.

In our opinion, the clinical management of AM is still challenging. Indeed, even if melanoma is responsible for a reduced percentage of all cutaneous cancers, it accounts for most deaths from skin neoplasms. Fortunately, recent knowledge on melanoma has led to the development of targeted therapies, which has changed the treatment landscape. In particular, drugs targeting immunological pathways or driver mutations and genetically engineered vaccines and viruses showed promising results in terms of efficacy. Moreover, the combination of different drugs with different mechanisms of action has overcome melanoma immune escape and resistance. However, in the decision-making on the appropriate adjuvant or neo-adjuvant treatment for patients with AM, the safety profile, and medical history of the patient should play a key role as well.

Certainly, further studies are needed in order to offer patients with AM a tailored-tail approach, which will increase survival while reducing the possibilities of side effects. The right drug at the right time and for the right patient should be the goal of the therapeutic management for patients with metastatic and unresectable melanoma.

## Figures and Tables

**Table 1:** Efficacy and safety of drugs currently approved for the management of metastatic or unresectable melanoma.

Clinical Trial	Drug and Dosage	Patients	ORR	CR	PR	AE (%)	Laboratory Abnormalities
KEYNOTE-006	Group A: pembrolizumab 10 mg/kg Q2W Group B: pembrolizumab 10 mg/kg Q3W Group C: ipilimumab 3 mg/kg Q3W	Group A: 277 Group B: 279 Group C: 278	Group A: 33% Group B: 34% Group C: 12%	Group A: 6% Group B: 5% Group C: 1%	Group A: 27% Group B: 29% Group C: 10%	Pembrolizumab: fatigue (28); rash (24); arthralgia (18). Ipilimumab: fatigue (28); rash (23); headache (14); decreased appetite (14).	Pembrolizumab: hyperglycemia (45); hypertriglyceridemia (43); anemia (35). Ipilimumab: hyperglycemia (45); anemia (33); hypertriglyceridemia (31).
KEYNOTE-002	Group A: pembrolizumab 2 mg/kg Q3W Group B: pembrolizumab 10 mg/kg Q3W Group C: chemotherapy	Group A: 180 Group B: 181 Group C: 179	Group A: 21% Group B: 25% Group C: 4%	Group A: 2% Group B: 3% Group C: 0%	Group A: 19% Group B: 23% Group C: 4%	Pembrolizumab: pruritus (28); rash (24); constipation (22). Chemotherapy: constipation (20); diarrhea (20); cough (16).	Pembrolizumab: hyperglycemia (49); hypoalbuminemia (37); hyponatremia (37). Ipilimumab: hyperglycemia (44); hypoalbuminemia (33); hypertriglyceridemia (32).
CHECKMATE-037	Group A: nivolumab 3 mg/kg Q2W Group B: chemotherapy	Group A: 268 Group B: 102	Group A: 32% Group B: NR	Group A: 3% Group B: NR	Group A: 28% Group B: NR	Group A: rash (21); pruritus (19); cough (17). Group B: rash (7); cough (6); peripheral edema (5).	Group A: increased AST (28); hyponatremia (25); increased alkaline phosphatase (22). Group B: hyponatremia (18); increased alkaline phosphatase (13); increased AST (12).
CHECKMATE-066	Group A: nivolumab 3 mg/kg Q2W Group B: chemotherapy	Group A: 210 Group B: 208	Group A: 34% Group B: 9%	Group A: 4% Group B: 1%	Group A: 30% Group B: 8%	Group A: fatigue (49); musculoskeletal pain (32); rash (28). Group B: fatigue (29); musculoskeletal pain (25); rash (12); pruritus (12).	Group A: increased ALT (25); increased AST (24); increased alkaline phosphatase (21). Group B: increased ALT (19); increased AST (19); increased alkaline phosphatase (14).
CHECKMATE-067	Group A: nivolumab 1 mg/kg + ipilimumab 3 mg/kg Q3W for 4 doses, followed by nivolumab 3 mg/kg Q2W Group B: nivolumab 3 mg/kg Q2W Group C: ipilimumab 3 mg/kg Q3W for 4 doses followed by placebo Q2W	Group A: 314 Group B: 316 Group C: 315	Group A: 50% Group B: 40% Group C: 14%	Group A: 9% Group B: 9% Group C: 2%	Group A: 41% Group B: 31% Group C: 12%	Group A: fatigue (62); diarrhea (54); rash (53). Group B: fatigue (59); rash (40); diarrhea (36). Group C: fatigue (51); diarrhea (47); rash (42).	Group A: increased ALT (55); hyperglycemia (53); increased AST (52); anemia (52). Group B: hyperglycemia (46); anemia (41); lymphopenia (41). Group C: anemia (41); lymphopenia (29); increased ALT (29); increased AST (29).
MDX010-20	Group A: ipilimumab 3 mg/kg Q3W + gp100 Q3W for 4 doses Group B: ipilimumab 3 mg/kg Q3W for 4 doses Group C: gp100 for 4 doses	Group A: 403 Group B: 137 Group C: 136	Group A: 6% Group B: 11% Group C: 2%	NA	NA	Group A: diarrhea (37); fatigue (34); rash (25). Group B: fatigue (41); diarrhea (32); pruritus (31). Group C: fatigue (31); diarrhea (20); pruritus (8).	Group A: NR Group B: NR Group C: NR
TRIAL 1	Group A: vemurafenib 960 mg twice a day Group B: dacarbazine 1000 mg/m^2^ Q3W	Group A: 337 Group B: 338	Group A: NR Group B: NR	Group A: NR Group B: NR	Group A: NR Group B: NR	Group A: arthralgia (53), alopecia (45), rash (37). Group B: nausea (43), fatigue (33), vomiting (26).	Group A: NR Group B: NR
TRIAL 2	Vemurafenib 960 mg twice a day	132	52%	2%	50%	Arthralgia (67), fatigue (54), rash (52).	NR
TRIAL 3	Group A: vemurafenib 960 mg twice a day Group B: vemurafenib 960 mg twice a day Group A: no prior local therapy for brain metastases. Cohort B: at least one local therapy.	Group A: 90 Group B: 56	Group A: 18% Group B: 18%	Group A: 2% Group B: 0%	Group A: 16% Group B: 18%	Group A: NR Group B: NR	Group A: NR Group B: NR
BREAK-3	Group A: dabrafenib 150 mg twice a day Group B: dacarbazine 1000 mg/m2 Q3W	Group A: 187 Group B: 63	Group A: 52% Group B: 17%	Group A: 3% Group B: 0%	Group A: 48% Group B: 17%	Group A: hyperkeratosis (37), headache (32), pyrexia (28). Group B: constipation (14), pyrexia (10), headache (8).	Group A: hyperglycemia (50), hypophosphatemia (37), increased alkaline phosphatase (19). Group B: hyperglycemia (43), hypophosphatemia (14), increased alkaline phosphatase (14).
Metastatic brain melanoma	Group A: dabrafenib 150 mg twice a day Group B: dabrafenib 150 mg twice a day Group A: no prior local therapy for brain metastases. Cohort B: at least one local therapy.	Group A: 74 Group B: 65	Group A: 18% Group B: 18%	NR	NR	Group A: NR Group B: NR	Group A: NR Group B: NR
COMBI-d	Group A: dabrafenib 150 mg twice a day + trametinib 2 mg once daily Group B: dabrafenib 150 mg twice a day + placebo	Group A: 211 Group B: 212	Group A: 66 Group B: 51	Group A: 10 Group B: 8	Group A: 56 Group B: 42	Group A: pyrexia (54), rash (32), chills (31). Group B: pyrexia (33), arthralgia (31), headache (30).	Group A: hyperglycemia (65), increased blood alkaline phosphatase (50), hypophosphatemia (38). Group B: hyperglycemia (57), hypophosphatemia (35), increased blood alkaline phosphatase (25).
COMBI-v	Group A: dabrafenib 150 mg twice a day + trametinib 2 mg once daily Group B: vemurafenib 960 mg twice a day	Group A: 352 Group B: 352	Group A: 64 Group B: 51	Group A: 13 Group B: 8	Group A: 51 Group B: 43	Group A: NR Group B: NR	Group A: NR Group B: NR
COLUMBUS CMEK162B2301, Part 1	Group A: encorafenib 450 mg once daily + binimetinib 45 mg twice daily Group B: encorafenib 300 mg once daily Group C: vemurafenib 960 mg twice daily	Group A: 192 Group B: 194 Group C: 191	Group A: 63% Group B: 51% Group C: 40%	Group A: 8% Group B: 5% Group C: 6%	Group A: 55% Group B: 45% Group C: 35%	Group A: fatigue (43), nausea (41), vomiting (30). Group B: NR Group C: rash (53), hyperkeratosis (49), fatigue (46), arthralgia (46).	Group A: increased creatinine (93), increased gamma glutamyl transferase (45), anemia (36). Group B: NR Group C: increased creatinine (92), increased alkaline phosphatase (35), increased gamma glutamyl transferase (34), anemia (34).
CMEK162B2301, Part 2	Group A: encorafenib 300 mg once daily + binimetinib 45 mg twice daily Group B: encorafenib 300 mg once daily	Group A: 258 Group B: 280	Group A: 66% Group B: 50%	Group A: NR Group B: NR	Group A: NR Group B: NR	Group A: NR Group B:NR	Group A: NR Group B: NR

**Table 2:** Efficacy and safety of drugs currently approved as the adjuvant treatment of patients with melanoma and lymph node involvement.

Clinical Trial	Drug and Dosage	Patients	Patients with Event	AE (%)	Laboratory Abnormalities
KEYNOTE-054	Group A: pembrolizumab 200 mg Q3W Group B: placebo	Group A: 514 Group B: 505	Group A: 26% Group B: 43%	Group A: diarrhea (28); pruritus (19); arthralgia (16). Group B: diarrhea (26); nausea (15); arthralgia (14).	Group A: increased ALT (27); increased AST (24); lymphopenia (24). Group B: increased ALT (16); lymphopenia (16); increased AST (15).
CHECKMATE-238	Group A: nivolumab 3 mg/kg Q2W Group B: ipilimumab 10 mg/kg Q3W 4 doses then every 12 weeks beginning at Week 24 for up to 1 year	Group A: 453 Group B: 453	Group A: 34% Group B: 46%	Group A: fatigue (57); diarrhea (37); rash (35). Group B: fatigue (55); diarrhea (55); rash (47).	Group A: lymphopenia (27); anemia (26); increased lipase (25); increased ALT (25). Group B: increased ALT (40); anemia (34); increased AST (33).
CA184-029	Group A: ipilimumab 10 mg/kg Q3W or 4 doses, followed by YERVOY 10 mg/kg or placebo every 12 week Group B: placebo	Group A: 475 Group B: 476	Group A: 49% Group B: 62%	Group A: rash (50); diarrhea (49); fatigue (46), Group B: fatigue (38); diarrhea (30); rash (20).	Group A: increased ALT (46); increased AST (38); increased lipase (26). Group B: increased lipase (17); increased ALT (16); increased AST (14).
COMBI-AD	Group A: dabrafenib 150 mg twice a day + trametinib 2 mg once daily Group B: placebo	Group A: 438 Group B: 432	Group A: 38% Group B: 57%	Group A: pyrexia (63), fatigue (59), nausea (40). Group B: fatigue (37), headache (24), nausea (20).	Group A: hyperglycemia (63), increased AST (57), increased ALT (48). Group B: hyperglycemia (47), increased ALT (18), neutropenia (12).

## Data Availability

Data sharing not applicable to this article as no datasets were generated or analyzed in the current study.
